# Novel bi-allelic variants expand the *SPTBN4*-related genetic and phenotypic spectrum

**DOI:** 10.1038/s41431-021-00846-5

**Published:** 2021-03-26

**Authors:** Markus Buelow, David Süßmuth, Laurie D. Smith, Omid Aryani, Claudia Castiglioni, Werner Stenzel, Enrico Bertini, Markus Schuelke, Ellen Knierim

**Affiliations:** 1 Department of Neuropediatrics, Charité – Universitätsmedizin Berlin, corporate member of Freie Universität Berlin und Humboldt Universität zu Berlin, Berlin, Germany; 2 HELIOS Kliniken – Helios Klinikum Hohenstücken, Berlin, Germany; 3 Department of Pediatrics, Division of Pediatric Genetics and Metabolism, The University of North Carolina SOM, North Carolina, NC USA; 4 Department of Neuroscience, Iranian University of Medical Sciences, Tehran, Iran; 5 Pediatric Neurology, Clínica Las Condes, Santiago, Chile; 6 Department of Neuropathology, Charité – Universitätsmedizin Berlin, corporate member of Freie Universität Berlin und Humboldt Universität zu Berlin, Berlin, Germany; 7 Unit of Neuromuscular and Neurodegenerative Disorders, Bambino Gesù Children’s Research Hospital, IRCCS, Rome, Italy; 8 NeuroCure Clinical Research Center, Charité – Universitätsmedizin Berlin, corporate member of Freie Universität Berlin und Humboldt Universität zu Berlin, Berlin, Germany

**Keywords:** Genetics research, Disease genetics

## Abstract

Neurodevelopmental disorder with hypotonia, neuropathy, and deafness (NEDHND, OMIM #617519) is an autosomal recessive disease caused by homozygous or compound heterozygous variants in *SPTBN4* coding for type 4 βIV-spectrin, a non-erythrocytic member of the β-spectrin family. Variants in *SPTBN4* disrupt the cytoskeletal machinery that controls proper localization of ion channels and the function of axonal domains, thereby generating severe neurological dysfunction. We set out to analyze the genetic causes and describe the clinical spectrum of suspected cases of NEDHND. Variant screening was done by whole exome sequencing; clinical phenotypes were described according to the human phenotype ontology, and histochemical analysis was performed with disease-specific antibodies. We report four families with five patients harboring novel homozygous and compound heterozygous *SPTBN4* variants, amongst them a multi-exon deletion of *SPTBN4*. All patients presented with the key features of NEDHND; severe muscular hypotonia, dysphagia, absent speech, gross motor, and mental retardation. Additional symptoms comprised horizontal nystagmus, epileptiform discharges in EEG without manifest seizures, and choreoathetosis. Muscle histology revealed both characteristics of myopathy and of neuropathy. This report expands the *SPTBN4* variant spectrum, highlights the spectrum of morphological phenotypes of NEDHND-patients, and reveals clinical similarities between the NEDHND, non-5q SMA, and congenital myopathies.

## Introduction

Spectrins are cytoskeletal proteins found in a variety of tissues and cell types. They were initially identified in erythrocytes [1]. Vertebrate spectrin is a hetero-tetramer formed by two α- and two β-subunits. The α-spectrin subgroup has two members (I and II), while the β-spectrin subgroup has five members (I–V). βIV-spectrin is enriched in the myelinated neurons of the central nervous system [2], where it has a role in the clustering of sodium and potassium channels at the axon initial segment (AIS) and at the nodes of Ranvier *via* interaction with ankyrin-G [3]. The *SPTBN4* gene encodes a non-erythrocytic βIV-spectrin. We published the first case of autosomal recessive myopathy caused by a homozygous pathogenic *SPTBN4* variant in 2017 [4]. The proband was boy of Kurdish descent who, in addition to the classical symptoms of myopathy, exhibited sensorineural hearing loss, intellectual disability, and Spectrin-associated neuropathy. A recent report by Wang et al. [5] described six additional children with this disease due to autosomal recessive *SPTBN4* variants. They demonstrated that a loss-of-function variant disrupted sodium and potassium channel clustering, leading to neuropathy. Häusler et al. reported a novel homozygous splice-site variant in a pair of siblings presenting with axonal neuropathy in the absence of intellectual disability [6]. In pigs, deletions in *SPTBN4* cause severe myopathy [7]. We now report five additional affected individuals from four families who were found to harbor variants that affect function of SPTBN4. Our findings further define the clinical spectrum of neurodevelopmental disorder with hypotonia, neuropathy, and deafness.

## Materials and methods

### Patients

The patients’ parents provided written informed consent for study participation, including publication of patient photographs, in accordance with the Declaration of Helsinki (Charité Ethics Committee approval EA2/107/14). Patients 1 and 2 were admitted to the Helios Klinikum Hohenstücken in Berlin, Germany, for neuro-rehabilitation; Patient 3 presented as an outpatient at UNC Chapel Hill’s Division of Pediatric Genetics and Metabolism, Chapel Hill, NC, USA; Patient 4 presented as an outpatient at the Iranian University of Medical Sciences, Tehran, Iran; and Patient 5 was treated and diagnosed at the Clínica Las Condes, Pediatric Neurology, Santiago, Chile.

### Sequencing and segregation analysis

Genomic DNA was extracted from white blood cells and whole exome sequencing (WES) was performed for all patients. Variant interpretation was performed according to current ACMG guidelines for variant classification [8]. We submitted our variants to ClinVar (VCV000987745, VCV0009877446, VCV000987747, VCV000988586).

### Histology, immunohistochemistry (IHC), and morphometry

Fiber-type-specific atrophy was measured by determining the minimal Feret’s diameter of each fiber from muscle cross-sections using a standardized method. The use of the minimal Feret’s diameter minimizes the confounding factor of oblique sectioning. For calculating the minimal Feret’s diameter all fibers of one representative 200× field of view of a ATPase pH 4.3 stained muscle section were counted. This amounted to *n* = 131 type 1 and *n* = 156 type 2 fibers in the patient, and *n* = 177 type 1 and *n* = 174 type 2 fibers in the control. IHC staining and imaging was performed as previously described [4]. Images were loaded into ImageJ (https://imagej.nih.gov/ij/) processing software, the circumference of individual fibers was manually traced and the minimal Feret’s diameter calculated using the build-in measuring algorithm. Values were visualized as cumulative histograms in GraphPad Prism (GraphPad Software Inc., San Diego, CA, USA).

## Results

### Clinical reports

#### Family A (Patients 1 and 2)

**Fig. 1:**
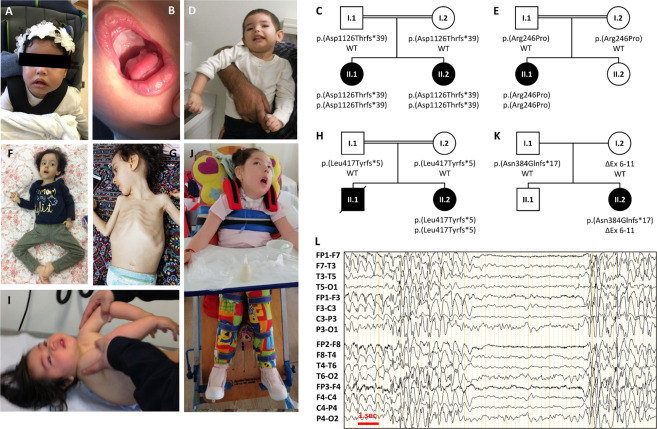
Clinical images of the patients and family trees. **A** Patient 2 (II.2) from Family A at age 2.5 years. **B** Note the tented upper lip vermillion, her mask-like facies, and the high palate. **C** Pedigree of Family A. **D** Patient 3 (II.2) from Family B at age 5. Note the lack of head control, choreoathetoid arm movements, and muscular hypotonia causing slip through when held in vertical position under the armpits. **E** Pedigree of Family B. **F**, **G** Patient 4 (II.2) from Family C at age 4 years. Note severe dystrophy due to feeding difficulties and unavailability of gastric tube feeding, myopathic facies, and severe muscle weakness with frog-leg posture. Her brother (II.1) passed away from aspiration pneumonia and had a similar phenotype. **H** Pedigree of Family C. **I**, **J** Patient 5 from Family D, **I** shows the severe muscle hypotonia, **J** depicts the same patient at 6 years not able to stand independently. **K** Pedigree of Family D. **L** EEG from Patient 5 at age 6 years shows generalized epileptic activity. No clinical seizures were observed in a 24-h video EEG recording. The parents had never observed any type of fits or seizures.

Two affected sisters were born at term to healthy first-cousin parents from Saudi Arabia. The pregnancy with Patient 1 was complicated by gestational diabetes. Muscular hypotonia was noted at birth. Feeding problems became apparent during the newborn period. She suffered from recurrent aspiration pneumonia and dysphagia from the first month of life, requiring gavage feeding from 1 year of age. Motor development was severely delayed, and the patient did not achieve early developmental milestones such as head control, rolling over, and crawling (Table 1). A physical examination at age 3 years revealed a high palate, myopathic facies, severe distal muscle weakness, generalized amyotrophy, bilateral ankle flexion contractures (Fig. 1A, B), and severe global developmental delay. Patient 1 was unable to stand, sit, eat, or drink without support at age 3 years. Her speech was limited to repeating single words in Arabic and German. Neurometabolic screening tests (tandem mass spectrometry of amino and organic acids, lactate, ammonia) and first-line genetic analyses (karyotyping, muscular dystrophy gene panel) were inconclusive. Serum CPK levels were normal. Cranial MRI revealed a diffuse T_2_-hyperintensity, predominantly affecting the subcortical white matter. Structural abnormalities were ruled out. MR spectroscopy was normal. A muscle biopsy was not performed. The younger sister of the patient (Patient 2) presented in a similar way but was more severely affected at the age of 2 years.

**Table 1:** Genetic and clinical features of our patients with *SPTBN4* variants. Symptoms are encoded according to Human Phenotype Ontology (HPO) [14].

	Characteristics and symptoms	HPO	Pat 1, Fam A (this report)	Pat 2, Fam A (this report)	Pat 3, Fam B (this report)	Pat 4, Fam C (this report)	Pat 5, Fam D (this report)
Mutation in *SPTBN4* (NM_020971)			c.3375_3393del p.(Asp1126Thrfs*39)	c.3375_3393del p.(Asp1126Thrfs*39)	c.737G>C p.(Arg246Pro)	c.1247del p.(Leu417Tyrfs*5)	c.1149dup p.(Asn384Glnfs*17) / g.(?_41,001,394)_(41,011,375_?)del
ACMG variant classification			PVS1	PVS1	PM2	PVS1	PVS1
Ethnicity			Saudi Arabia	Saudi Arabia	Afghanistan	Iran (Kurdish)	Chile (Latin American)
Gender			Female	Female	Female	Female	Female
Consanguinity			Y	Y	Y	Y	N
Zygosity			Hom	Hom	Hom	Hom	Comp Het
Head							
	Myopathic facies	HP:0002058	Y	Y	Y	Y	Y
	Poor head control	HP:0002421	Y	Y	Y	Y	Y
	High palate	HP:0000218	Y	Y	Y	Y	N
	Sensorineural hearing impairment	HP:0000407	N (clinically)	N (clinically)	Y	N (clinically)	Y
	Absent brainstem auditory responses	HP:0004463	ND	ND	Y	ND	N
	Scoliosis	HP:0002650	N	N	N	Y	Y
Respiratory and chest							
	Recurrent infections due to aspiration	HP:0004891	Y	Y	Y	Y	Y
Gastronintestinal							
	Feeding difficulties	HP:0011968	Y	Y	Y	Y	Y
	Poor suck	HP:0002033	Y	Y	Y	Y	Y
	Dysphagia	HP:0002015	Y	Y	Y	Y	Y
	Gastroesophageal reflux	HP:0002020	U	U	Y	Y	Y
	Gastrostomy tube feeding in infancy	HP:0011471	Y	Y	Y	N	Y
Skeletal							
	Ankle contracture	HP:0006466	N	N	N	Y	Y
Neurologic							
	Neonatal hypotonia	HP:0001319	Y	Y	Y	Y	Y
	Generalized hypotonia	HP:0001290	Y	Y	Y	Y	Y
	Generalized muscle atrophy	HP:0009055	Y	Y	N	Y	Y
	Distal limb muscle atrophy	HP:0003693	Y	Y	N	Y	Y
	Choreoathetoid movements	HP:0001266	N	N	Y	N	N
	Abnormality of the cerebral white matter	HP:0002500	Y	ND	N	ND	N
	Demyelinating peripheral neuropathy	HP:0003448	N	N	N	N	N
	Peripheral axonal neuropathy	HP:0003477	N	N	N	N	Y
	Type 1 muscle fiber atrophy	HP:0011807	ND	ND	Y	ND	Y
	Type 2 muscle fiber atrophy	HP:0003554	ND	ND	N	ND	Y
	Areflexia	HP:0001284	Y	Y	N	Y	Y
	Delayed gross motor development	HP:0002194	Y	Y	Y	Y	Y
	Horizontal nystagmus	HP:0000666	N	N	Y	Y	N
	EEG abnormality	HP:0002353	ND	ND	Y	ND	Y
	Absent speech	HP:0001344	Y	Y	Y	Y	Y
Prenatal manifestation							
	Premature birth (<37 weeks of gestation)	HP:0001622	N	N	N	N	N

#### Family B (Patient 3)

Patient 3 is a 5-year-old female and the first child born to healthy paternal second cousins from Afghanistan. Pregnancy and birth were normal. Muscle hypotonia was diagnosed in the neonatal period. She suffered from recurrent pulmonary aspiration starting at 5–6 months of age. She had developed neither head control, independent sitting, crawling, nor talking by 2 years of age. A physical exam revealed horizontal nystagmus, choreoathetosis of the arms with intermittent dystonia, and generalized hypotonia (Fig. 1D). Deep tendon reflexes were brisk without clonus. Laboratory testing for inborn errors of metabolism, routine karyotyping, and microarray analysis were  inconclusive. Cranial MRI and CT were normal. MRS revealed nonspecific lipid and lactate peaks with increased glutamine/glutamate peaks in the region of the basal ganglia and the *Corpus callosum*. Oral feeding led to recurrent aspiration pneumonias and dystrophy with a body weight below the first percentile. Gastrostomy feeding was started at 3 years of age. Seventy-two-hour EEG showed abundant to nearly continuous centro-parietal sharp/spike/poly-spike wave discharges during sleep without clinical correlation to manifest seizures. Nerve conduction studies and EMG were entirely normal. Otoacoustic emissions were present. In brainstem evoked response audiometry (BERA) only wave 1 from the inner ear could be recorded pointing to a defect at the level of cochlear nerve conduction.

#### Family C (Patient 4)

Patient 4 is a 4-year-old female born to healthy consanguineous parents from Iran with Kurdish background. Her older brother died at age 2 years due to increasing feeding difficulties and subsequent aspiration pneumonia. His phenotype was described as similar. Patient 4 showed signs of general hypotonia and muscle weakness shortly after birth (Fig. 1F). Routine metabolic testing, including acylcarnitine and urine organic acids, was normal. Genetic testing for spinal muscular atrophy type 1 was negative. She had poor head control and neither sat, crawled, nor spoke, and had severe dystrophy (Fig. 1G). She presented with clinical signs of myopathy, including myopathic facies, high arched palate, and bilateral ankle flexion contractures. She had horizontal nystagmus. EEG or BERA were not recorded. Sensory-nerve action potentials and motor action potentials were normal.

#### Family D (Patient 5)

Patient 5 is a 7-year-old girl born to healthy non-consanguineous parents from Chile. She was born at term, following a normal pregnancy and delivery. Her parents noted a weak suck and slow weight gain during her first week of life. Muscular hypotonia, motor delay, and frequent choking and gagging while feeding were noted at 3 months of age. Severe gastroesophageal reflux with nasal regurgitation led to recurrent respiratory infections. Patient 5 had attained only partial head control and could neither sit nor stand (Fig. 1I, J). An examination at 10 months of age showed mild weakness of facial musculature, severe hypotonia without trunk control, bilateral *Talipes equinovarus* and *Pes cavus*, and absent deep tendon reflexes. Though able to exert spontaneous antigravity movements of the upper and lower limbs, she was unable to lift her head when prone. Bilateral moderate to severe hearing loss was diagnosed at 18 months of age, at 3 years her speech recognition threshold was 80 dB HL. Her growth parameters at age 4 years were below the third percentile. Tracheostomy and gastrostomy were required due to increasing dysphagia and a weak cough reflex coupled with recurrent pneumonias. Karyotyping, serum CPK levels, Prader–Willi syndrome (tested by methylation analysis), and spinal muscular atrophy type 1 genetic testing were normal. cMRI did not show any abnormalities. Nerve conduction studies showed normal sensory and motor conduction velocities, with low-amplitude motor responses. EMG showed signs of acute and chronic denervation such as fibrillation potentials and positive sharp waves, motor unit potentials with increased amplitude and duration, and decreased recruitment. An EEG at age 6 years revealed almost continuous and severe generalized epileptiform activity without any clinical correlate of manifest seizures (Fig. 1L).

### Whole exome sequencing identifies variants in *SPTBN4*

WES of the patients and their parents revealed novel bi-allelic variants in *SPTBN4* in all four families. Deletions were found in Families A and C. Patient 1 and 2 from Family A were homozygous for [chr19:g.41,025,779_41,025,797del (hg19); NM_020971.3c.3375_3393del; p.(Asp1126Thrfs*39)] (Fig. 1C) and Patient 4 from Family C was homozygous for [chr19:g.41,008,725_41,008,725del (GRCh37); NM_020971.3c.1247del; p.(Leu417Tyrfs*5)]. These deletions cause frameshifts that lead to a premature stop codon. Sanger sequencing was done in Family A and verified the variant and its segregation. Both parents were heterozygous. Patient 3 from Family B was homozygous for the missense variant [chr19:g.41,003,464 (GRCh37); NM_020971.3c.737G>C; p.(Arg246Pro)] (Fig. 1E) that was absent from gnomAD. Patient 5 from Family D was compound heterozygous for two variants. The paternally inherited single base insertion [chr19:g.41,008,360dup (GRCh37); NM_020971.3c.1149dup; p.(Asn384Glnfs*17)] causes a frameshift that leads to a subsequent premature stop codon. The maternally inherited deletion with breakpoint spanning [chr19.g.(?_41,001,394)_(41,011,375_?)del (GRCh37)] encompasses exons 6–11 of *SPTBN4* (Fig. 1K).

### Histology and morphometry

**Fig. 2:**
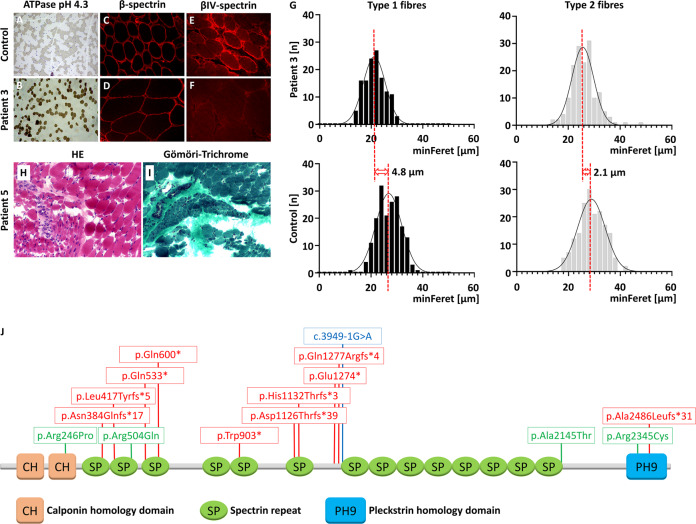
Histological and morphometric investigations. **A**, **B** ATPase pH 4.3 staining showing type 1 fiber (dark) hypotrophy in Patient 3 (**B**) in comparison to an age-matched control (**A**) (200×, scale bar 50 µm). Immunostaining of the same samples with an antibody directed against β-spectrin showed staining of the sarcolemma in both individuals (**C**, **D**, 600× and 200×, respectively). Staining for βIV-spectrin did not show any signal at the sarcolemmal position of the Patient 3 (**F**) in contrast to a healthy control (**E**) (400×). **G** Histogram of diameters from type 1 and type 2 muscle fibers from Patient 3 and an age-matched control showing an over-proportional thinning of type 1 fibers. **H** and **I** neurogenic changes with atrophic muscle fascicles and angulated fibers next to normally appearing muscle fibers in HE and Gömori-trichrome staining. **I**. **J** Summary of so far published and here described disease-causing variants in *SPTBN4*. NB, the multi-exon deletion is not marked on the graph.

Muscle biopsy was performed in Patients 3 and 5. In Patient 3, ATPase pH 4.3 staining and IHC with an antibody directed against myosin heavy chain slow (MHC neonatal, NCL-MHCn, Novocastra, 1:20) revealed a reduction of type 1 fiber diameters but no manifest fiber-type disproportion (Fig. 2A, B). Both fiber types were nearly equally abundant in age-matched controls of the vastus lateralis muscles. Consistent with these findings, morphometric analysis of muscle fibers from Patient 3 showed that type 1 fiber diameter reduction was higher than in type 2 fibers, (Feret diameter: type 1 = 21.0 ± 3.8 µm versus type 2 = 25.6 ± 4.9 µm), a finding that we know from patients with congenital myopathies, whereas in control muscle (Feret diameter: type 1 = 26.3 ± 4.5 µm versus type 2 = 28.3 ± 5.0 µm) the difference was significantly smaller. There were no signs of increased fiber diameter variability. Signs of denervation, such as atrophic angulated fibers or fiber grouping, were not observed (Fig. 2G).

In contrast to these findings, H&E/Gömöri-trichrome staining of a muscle biopsy sample from Patient 5 showed clear signs of neurogenic changes with atrophic fascicles, dark fibers (type 1) as well as brighter fibers (type 2) small and angulated fibers next to populations of muscle fibers with preserved diameter and groups of small fibers (Fig. 2H, I). ATPase activity at pH 9.4 showed normal fiber differentiation, atrophic fibers were of type 1 and 2 with a similar proportion of both types and groups of both types (not shown).

For IHC we had cryopreserved muscle tissue only from Patient 3 at our disposal. IHC with an antibody directed against β-spectrin (NCL SPEC1, clone RB C2/3D5, Novocastra UK; 1:100) showed a strong sarcolemmal demarcation of muscle fibers in Patient 3 and an age-matched control (Fig. 2C, D). In contrast, IHC with an antibody directed against the non-erythrocytic βIV-spectrin subtype 4 (sc-368195, H-85, Santa-Cruz, 1:100) showed no staining in Patient 3, while staining was preserved in age-matched control muscle (Fig. 2E, F).

## Discussion

Spectrins are molecular scaffold proteins that link the plasma membrane to the actin cytoskeleton. They are crucial for the determination of cell shape, arrangement of transmembrane proteins, and organelle organization. Variants in *SPTBN4* result in ion-channel dysfunction by disrupting the cytoskeletal machinery that controls proper localization of channels and the function of axonal domains in the AIS and in the nodes of Ranvier, where axonal ion channels are normally clustered causing a variety of nervous system dysfunctions.

We have identified multiple novel variants that affect function of *SPTBN4* in patients with severe muscular hypotonia, dysphagia, absent speech, delayed gross motor development, and intellectual disability. These symptoms are considered key features of the *SPTBN4*-related disorder. We have broadened the disorder’s clinical spectrum by describing the variably present features of areflexia, axonal motor neuropathy, nystagmus, epileptiform activity in EEG without clinical correlation, and a movement disorders with choreoathetosis.

Analysis of muscle biopsy specimens from affected infants revealed signs of a primary myopathy as well as of secondary neuropathic features. Electrophysiology revealed signs of obvious neuropathy only in Patient 5. This was in accordance with the neuropathic pattern characterized by neurogenic fiber-type grouping in the histopathological studies for this patient. βIV-spectrin was absent from the sarcolemma of Patient 3, while ATPase staining showed hypotrophic type 1 fibers, but no clear fiber-type disproportion, a finding more characteristic for congenital myopathies. We hypothesize that βIV-spectrin deficiency directly impacts the structural stability of the sarcolemma and the initiation or propagation of the depolarization waves along the myofiber and its T-tubular system. We derived that the evidence for a myopathy is mostly from the clinical and histopatholocigal findings, but not from functional studies about the role of SPTBN4 in muscle cells. Further studies are thus needed to determine the impact of pathogenic *SPTBN4* variants on the muscle cells proper. Our clinical phenotyping and neurophysiological studies suggest that the muscle weakness seen in patients with *SPTBN4* disorder may be caused by a combination of axonal neuropathy and congenital myopathy. This does not rule out the possibility that one pathological principle may dominate as described by Wang et al. (2018), where neuropathy seemed to be the predominant feature.

Though two of our patients did not exhibit clinically manifest seizures, their  EEGs showed highly pathologic epileptiform discharges. As βIV-spectrin plays a role in the clustering of *KCNQ2* subunit-containing potassium channels, there could be some degree of overlap of clinical symptoms with early-infantile epileptic encephalopathy type 7 and with benign familial neonatal seizures type 1. This is supported by Wang et al. who reported that three of their six patients had clinically manifest epilepsy, with two patients being refractory to antiepileptic medication. As variants in *KCNQ2* cause a wide range of phenotypes even within single families with members sharing the same variant [9], we can extrapolate that this may also apply to a protein involved in KCNQ2 clustering. A review of all reported cases of *SPTBN4-*related disorders shows that epileptic activity and seizures are more common than initially thought. In contrast to the pair of siblings described by Häusler et al. [6], all our patients with a molecular diagnosis of *SPTBN4* disorder had severe intellectual disability, indicating that intellectual disability is frequent in this condition. This information must be taken into account when counseling patients and families with *SPTBN4* disorder.

We identified and described five additional cases with pathogenic bi-allelic *SPTBN4* variants. Four of these were novel and two resulted in a frameshift. The pathogenic homozygous missense variant identified in Patient 3 led the exchange of an evolutionary conserved proline for an arginine. This variant was absent in the gnomAD database and in ClinVar [10]. Patient 5 harbors a multi-exon deletion on the maternally inherited allele and a small insertion leading to a frameshift on the paternally inherited allele. This is the first description of a multi-exon deletion in the *SPTBN4* gene and it shows that larger *SPTBN4* deletions may account for a part of *SPTBN4-*related disorder. Screening for large genomic *SPTBN4* rearrangements should improve molecular diagnostic rates for this population, in particular for patients where only a single variant that affects function has been identified.

To date, including our report, 15 pathogenic variants—truncating (*n* = 10), missense (*n* = 4), splice-site (*n* = 1) variants, and multi-exon deletion (*n* = 1)—have been reported in *SPTBN4* [4–6, 11–13] (Fig. 2J). Most affected individuals reported to date are homozygous. In this report we present the first individual with a multi-exon deletion. Patients generally suffer from severe developmental delay and intellectual disability, although two individuals in one family had a milder phenotype, including one individual with normal cognitive development. Speech and language skills are often severely limited. Affected individuals rarely achieve head control and are unable to sit, stand, or walk. They typically have congenital muscular hypotonia. Axonal motor neuropathy leads to hyporeflexia/areflexia and weakness. Most affected individuals require tube feeding. More than half of them develop seizures or have a pathological EEG. The mutations are dispersed over the whole gene and we do not see a clear genotype–phenotype correlation.

Our study further broadens the clinical and variant spectrum of congenital and early-onset *SPTBN4*-related disorder. With the accumulation of data on βIV-spectrinopathies, it seems rational that *SPTBN4* genetic testing should also be considered in patients with early-onset hypotonia, motor developmental delay, and intellectual disability, especially in the presence of axonal neuropathy, deafness, or pathological discharge patterns on the EEG.
